# Multisectoral approaches to the prevention and control of vector-borne diseases: lessons learned from case studies

**DOI:** 10.1186/s13071-025-07181-4

**Published:** 2025-12-06

**Authors:** João Bueno Nunes, Rodrigo Gurgel-Gonçalves, Vanessa Resende Nogueira Cruvinel, Everton Nunes da Silva, Marcos Takashi Obara, Natália Oliveira Mota, Tara Rava Zolnikov, Gildas A. Yahouedo, Florence Fouque

**Affiliations:** 1 Laboratory for the Synthesis and Analysis of Biomolecules, Chemistry Institute, University of Brasília, Brasília, Brazil; 2 Laboratory of Medical Parasitology and Vector Biology, Faculty of Medicine, University of Brasília, Brasília, Brazil; 3 Faculty of Health Sciences and Technologies, University of Brasília, Brasília, Brazil; 4 School of Health Professions, National University, San Diego, CA USA; 5 UNICEF/UNDP/World Bank/WHO Special Programme for Research and Training in Tropical Diseases (TDR), World Health Organization, Geneva, Switzerland

**Keywords:** Multisectoral approach, Vector-borne diseases, Malaria, Arboviral diseases, Control

## Abstract

In this report, we summarize the main outputs of the final workshop on multisectoral approaches (MSAs) to vector-borne diseases (VBDs) supported by the Special Programme for Research and Training in Tropical Diseases. We discussed the results of case studies from different countries investigating the collaboration between sectors such as health, education, environment, agricultural, and water and sanitation, among others, to combat VBDs such as malaria and arboviral diseases. Over 4 days, principal investigators of case studies presented their outcomes and invited experts highlighted the importance of MSA for effective control of VBDs. This multisectoral approach is novel and adds value to the research field by using a comprehensive strategy through coordinated efforts to address difficult public health topics, like vector-borne disease prevention and treatment. The participants had the opportunity to brainstorm and propose solutions related to limited financial resources to support MSA activities, the difficulty of engaging and collaborating with stakeholders from nonhealth sectors, and the sustainability of engaging committees that work across disciplines. Discussions emphasized the necessity of MSA and the importance of innovative technologies and multidisciplinary research, including strategic solutions that can help countries develop, implement, and sustain MSA for the prevention and control of VBDs. The workshop concluded with a commitment to strengthen international partnerships and implement comprehensive MSA to effectively control VBDs in marginalized communities.

**Graphical Abstract:**

**Figure:**
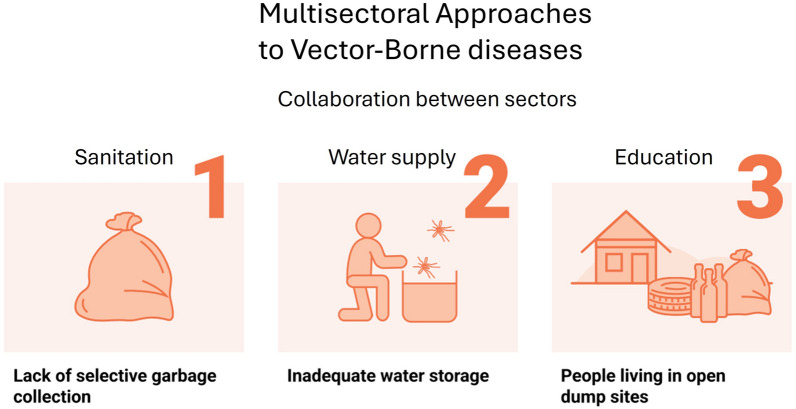

## Background

The World Health Organization (WHO) estimates that more than 17% of all infectious diseases are vector-borne diseases (VBDs) with more than 700,000 deaths annually [1]. Simultaneously, these diseases have the highest incidence in tropical and subtropical areas and drastically affect the poorest populations in these areas [2]. Mosquito-borne diseases such as dengue, chikungunya, Zika, urban yellow fever, and malaria are epidemics in the Americas and Africa, and their control is key to preventing deaths. *Aedes aegypti* is present in urban areas, and the number of dengue cases in the Americas has increased from 0.5 million in 1980 to 3 million in 2019. Brazil recently experienced its largest dengue epidemic in 2024, with over 6 million probable cases and 4000 confirmed deaths [3].

Active public and government engagement is necessary for controlling VBDs. In this integrated strategy, intersectoral actions are needed to plan, finance, and implement priority activities, as proposed in the multisectoral approach (MSA) to the prevention and control of VBDs [4, 5]. MSA involves the collaboration between stakeholders from the health sector and other nonhealth sectors, including government bodies, nongovernmental organizations, communities, and other stakeholders with common goals on specific health issues [6]. This approach recognizes that positive health outcomes cannot be achieved effectively and efficiently by the health sector alone, but require the involvement of other sectors, including but not limited to, environment, agriculture, economy, water and sanitation, and communities [7]. Many studies have shown the contribution of MSAs on health performance and outcomes, improving the well-being of populations [8–10].

To provide countries with the capacity to develop and implement MSA, the Special Programme for Research and Training in Tropical Diseases (TDR) cosponsored by the United Nations Children’s Fund (UNICEF), United Nations Development Programme, World Bank, and WHO led the development and release of a conceptual framework on MSA for the prevention and control of VBDs.^9^ TDR has also supported research institutions and national vector control programs, as well as case studies on MSA against arboviral diseases and malaria, and organized training workshops in collaboration with other WHO entities, including the Water, Sanitation, and Hygiene (WaSH) Unit, the Department of Control of Neglected Tropical Diseases, and the Global Malaria Programme. This MSA is novel and adds value to the research field by using a comprehensive strategy through coordinated efforts to address difficult public health topics, like vector-borne disease prevention and treatment.

In June 2024, the final TDR workshop on MSA for the prevention and control of VBDs was organized to present results from the supported case studies, to share experiences, and to promote collaboration between different sectors in combating diseases such as malaria, dengue, Zika, among others. The event brought together experts from various sectors, including health, environment, agriculture, education, and Water, sanitation and hygiene (WaSH), to share knowledge, experiences, and strategies for controlling VBDs through a multisectoral approach. In this report, we summarize the main outputs of the 4-day workshop, including plenary lectures, group discussions, and strategic solutions to support country efforts in the prevention and control of VBDs.

## Plenary lectures

The workshop took place in Brasília, Brazil, hosted by the University of Brasília. There were 54 participants: 30 Brazilians (55.6%) and 24 from other countries (44.4%), including Benin, Burkina Faso, China, Cuba, Ecuador, France, Kenya, the Netherlands, Nigeria, Peru, Portugal, Senegal, Switzerland, and the USA. The workshop opened with keynote lectures delivered by experts from different countries, covering key points such as (i) the importance of MSAs for effective disease control, (ii) the role of community engagement in enhancing disease control efforts, (iii) diverse environmental and agricultural factors influencing disease persistence, and (iv) the pivotal role of innovation and multidisciplinary approaches in advancing progress. The experts outlined the connections between the four categories of determinants impacting the transmission of VBDs across various sectors, emphasizing the necessity of MSA in addressing VBDs.

Integrated Vector Management (IVM) and MSA were recognized as essential approaches for addressing these complex and challenging interconnections and promoting sustainable disease control. The experts described how factors such as intensive agriculture (e.g., rice production), poor WaSH conditions, and climate change contribute significantly to the spread of VBDs. They emphasized the need for a renewed focus on WaSH to prevent and control mosquito-borne diseases. They pointed out that displaced populations typically experience lower healthcare access and highlighted climate change as a key driver contributing to population displacement. Proposed interventions involve strategies such as preventing the accumulation of stagnant water, ensuring proper covering and regularly cleaning and disinfecting of water storage containers, managing and removing unintended containers, and safely storing and disposing of domestic waste. Other presentations shed light on the need to address environmental and agricultural practices, such as reducing pesticide misuse, deforestation, and land mismanagement, to achieve effective control.

The urban cleaning sector highlighted the impact of solid and liquid waste management on the presence of mosquito aquatic breeding habitats and therefore on dengue control. It stressed the importance of community involvement in maintaining urban cleanliness and managing waste to effectively reduce dengue vector density and cases of diseases. Finally, the water sector highlighted the connection between promoting public health in Brazil and the quality of drinking water, linked to the management of solid and liquid waste. Engaging communities in public health initiatives, as shown in projects like Malakit (self-diagnosis and treatment of malaria in the Guianas countries) [2], and VIGIA (Peruvian Ministry of Health [MINSA] project on confronting the threats of emerging and re-emerging diseases).

Innovative tools like mobile apps for mosquito surveillance, the use of biopesticides, and targeted drug administration (e.g., the Curema project for *Plasmodium vivax* malaria control in the Guiana Shield region) were presented as critical advancements. Multidisciplinary research and the development of new funding mechanisms were emphasized as necessary to implement MSA and sustain progress in controlling VBDs. The latter plays a crucial role in promoting collaboration across different sectors. Other sectors were also found relevant in the fight against VBDs as reported from Brazil such as the mining sector. The mining sector shares its experiences on the relationships between mining activities and malaria cases in the Amazon, including the difficulties in controlling malaria among indigenous populations and miners in remote areas, especially between workers involved in illegal mining activities [11].

## Working groups

After the presentation sessions, the participants were split into two groups: one focusing on MSA for the control of Neglected Tropical Diseases (NTDs) and arboviral diseases (group A) and the other focusing on MSA for malaria control (group B). The groups were tasked with brainstorming three key challenges for a successful MSA: (i) how to interest nonhealth sectors to foster multisectoral collaboration, (ii) resource allocation to support activities, and (iii) sustainability of multisectoral coordination committees. Below are the summarized outputs.

## MSAs against NTDs and arboviral diseases

The working group recognized the importance of collaboration with nonhealth sectors, since the management of arboviral diseases cannot be addressed by the health sector alone. Some items were specified to encourage these collaborations. (i) mandatory legislation can stipulate which sectors must collaborate in the field of disease prevention. As an example, the collaboration of the public health sector with the WaSH sector is critical in the management of mosquito aquatic breeding habitats. (ii) Institutionalizing these collaborations means that multisectoral efforts are structured and operational irrespective of political leadership. (iii) Highlighting the broader benefits and how preventing these diseases will result in economic development, infrastructure resilience, and improved quality of life.

To effectively control arboviral diseases, resources should be strategically allocated to support essential activities. The following points were proposed by the working group. (iv) Establish a cross-funding mechanism, where various sectors combine resources to invest in critical interventions, such as VBD surveillance including preparedness, early warning, monitoring, and control. While challenging to implement, this approach would ensure sustained funding for fundamental programs. (v) Prioritize capacity building in community-based organizations to facilitate their participation in NTD control activities. (vi) Ensuring that coordination is practical and economically viable, through coordinated funding mechanisms, can enhance sectoral participation.

The working group reported that to achieve long-term success, a robust and lasting multisectoral coordination committee must be established: (i) Leadership’s unreliability requires alternative mechanisms for continuity. (ii) Implement a formal and institutionalized strategy for multisectoral collaboration. (iii) Strengthen consultations, collaborations, and follow-ups for sustaining sector involvement. (iv) Integrate both voluntary and paid participation guarantees enduring dedication. (v) Interventions should be tailored to local contexts, leveraging community insights rather than imposing solutions from the top down, to ensure effectiveness and sustainability.

## MSAs against malaria

The working group for MSAs against malaria control also stressed that it is not possible to control malaria through the activities of the health sector alone. The Ministry of Environment was found particularly important in reaching vulnerable groups because restricted access to indigenous communities remains a significant challenge, reducing the effectiveness of environmental management interventions. To address this, shared resources and transport materials are needed to enable effective outreach and intervention. The infrastructure sector must recognize its role in the prevention of malaria by investing in improved housing conditions for low-income groups. Their living conditions are currently favoring breeding sites for mosquitoes and increased transmission of malaria. To encourage greater involvement from nonhealth sectors in malaria control, the group emphasized the importance of demonstrating how participation would align with their priorities and advance their own mandates. They also advocated policies offering incentives to nonhealth sectors to engage in disease control efforts. The working group also recommended developing regulatory frameworks that mandate or encourage sectors such as construction and agriculture to support vector control measures. They also highlighted the improvement in community and/or workforce health and safety that would result from controlling malaria, which would benefit all sectors. Finally, the working group recommended establishing a link between disease control and environmental sustainability to appeal to sectors focusing on agriculture, water management, and urbanism. In the academic community, studies should focus on developing culturally appropriate communication strategies to effectively engage communities and bring about behavior change.

The working group noted that effective malaria control depends on the productive allocation of resources to critical activities, such as public programs and education courses, making sure that communities are fully aware of methods of prevention. However, they pointed out that bureaucratic challenges experienced by the Ministry of Health, for example, the complexities in licensing, are one of the biggest impediments to this effort. Eliminating such regulatory barriers would enable more effective utilization of resources and a faster rate of malaria control. Supporting and financing the nonhealth sectors engaging actively on malaria control will enhance the efficiency and durability of malaria control. Encouraging businesses to incorporate the control of VBDs into their Corporate Social Responsibility (CSR) initiatives can significantly benefit community well-being. Demonstrating the positive impact on community health can enhance a company’s brand image and foster a favorable reputation and increasing customer loyalty.

Finally, the group discussed sustainability, starting with the continuity of a multisectoral coordination committee. They emphasized that one of the biggest challenges is the lack of continuity in leadership, particularly in the Ministry of Health, where bureaucratic challenges tend to hinder progress. To overcome such challenges, they suggested a formal and institutionalized approach to cross-sectoral collaboration to guarantee that partnerships are not leader-dependent but institutionalized commitments, and continuing engagement with communities, as prevention against malaria involves behavior change in the long term and locally driven action.

## Conclusions

The final TDR workshop on MSAs highlighted the significant strides made for the control of VBDs through multisectoral collaboration, while also recognizing persistent challenges. Some challenges are contextual, and the engagement of nonhealth sectors depends on the disease and population group, as needs and requirements are not the same. The required flexibility of MSAs was well illustrated through the discussion in the working groups. Other challenges are more structural regardless of the disease and the populations, such as inadequate infrastructure and framework for collaboration, funding, coordination, incentives for engagement of nonhealth sectors, and unsustainable multisectoral coordination committees [10].

Continued efforts are needed at country levels to effectively involve nonhealth sectors, raise and secure funds for the implementation of a comprehensive MSAs engaging communities, strengthen regional and international partnerships for experience sharing, and sustain multisectoral coordination committees. Local capacity building is also required to better inform the development and implementation of any MSA project, ensuring full consideration of existing and new resources against VBDs, i.e., strategies, tools, and technologies.

## Data Availability

Data sharing is not applicable.

## Abbreviations

VBD: Vector-borne disease
TDR: Special Programme for Research and Training in Tropical Diseases
MSA: Multisectoral approach
UNICEF: United Nations Children’s Fund
UNDP: United Nations Development Programme
WHO: World Health Organization
IVM: Integrated Vector Management
WaSH: Water, Sanitation and Health
NTD: Neglected tropical diseases
